# Severe Acute Respiratory Syndrome Coronavirus-2 Delta Variant Study In Vitro and Vivo

**DOI:** 10.3390/cimb45010019

**Published:** 2022-12-30

**Authors:** Hranush Avagyan, Sona Hakobyan, Arpine Poghosyan, Lina Hakobyan, Liana Abroyan, Elena Karalova, Aida Avetisyan, Mariam Sargsyan, Bagrat Baghdasaryan, Nane Bayramyan, Diana Avetyan, Zaven Karalyan

**Affiliations:** 1 Laboratory of Cell Biology and Virology, Institute of Molecular Biology of NAS RA, 7 Hasratyan St, Yerevan 0014, Armenia; 2 Experimental Laboratory, Yerevan State Medical University, 2 Koryun St, Yerevan 0025, Armenia; 3 Department of Epidemiology and Parasitology, Armenian National Agrarian University, 74 Teryan St, Yerevan 0009, Armenia; 4 Laboratory of Human Genomics and Immunomics, Institute of Molecular Biology of NAS RA, 7 Hasratyan St, Yerevan 0014, Armenia; 5 Department of Medical Biology, Yerevan State Medical University, 2 Koryun St, Yerevan 0025, Armenia

**Keywords:** SARS-CoV-2, Syrian hamsters, pathogenesis

## Abstract

At the end of 2019, an outbreak of a new severe acute respiratory syndrome caused by a coronavirus occurred in Wuhan, China, after which the virus spread around the world. Here, we have described the adaptive capacity and pathogenesis of the SARS-CoV-2 Delta variant, which is widespread in Armenia, in vitro and vivo on Syrian hamsters. We have studied the changes in the SARS-CoV-2genome using viral RNA sequencing during virus adaptation in vitro and in vivo. Our findings revealed that SARS-CoV-2 in Syrian hamsters causes a short-term pulmonary form of the disease, the first symptoms appear within 48 h after infection, reach 5–7 days after infection, and begin to disappear by 7–9 days after infection. The virus induces pathogenesis in the blood and bone marrow, which generally corresponds to the manifestation of the inflammatory process. The pulmonary form of the disease passes faster than changes in blood cells and bone marrow. Our data show that hamster organs do not undergo significant pathological changes in the Delta variant of SARS-CoV-2 infection.

## 1. Introduction

At the end of 2019, an outbreak new severe acute respiratory syndrome caused by novel coronavirus occurred in Wuhan City, China, and after this virus spread rapidly in the world. Sever Acute Respiratory Syndrome Coronavirus-2 (SARS-CoV-2) is a highly transmissible and pathogenic coronavirus, belonging to the Sarbecovirus subgenus of the Betacoronavirus genus [1,2,3]. Soon after the detection of the virus, data on its adaptation to replication in vitro were published [3,4,5,6]. The virus adaptation process is the most important step towards obtaining tools that allows the study of cytopathogenesis, virus replication features, evolution of the virus, the study of antiviral drugs, etc.

Even though the SARS-CoV-2 Delta variant (B.1.617.2) emerged during India’s second wave of infections, Delta variants have grown dominant internationally and are still evolving [7]. In this aspect, we have carried out the adaptation of the Delta variant of SARS-CoV-2 widespread in Armenia to the Vero cellular culture. The main goals of our work were to describe mechanisms of SARS-CoV-2Delta strain adaptation, to identify the main characteristics of the cytopathogenesis of the adapted virus, changes in the genome of the virus in the process of its adaptation, the dynamics of virus replication in cell culture, etc.

The one of the best standard animal models for SARS-CoV-2 investigation in vivo is model on Syrian hamster [8,9,10]. Virus infected hamsters provide closely resemble clinical and histopathological findings as manifested in virus infection of humans. As shown in several articles Syrian hamsters under the virus infection develop interstitial pneumonia [9,11]. Many studies have reported other pathological changes in Syrian hamsters during the virus infection [12,13,14,15,16,17]. However, these studies do not provide information about immune system cells and virus changes at early stages of infection. Here, we refined further virus and host parameters to increase the value of this small animal model. First, we demonstrated changes in blood cell populations of SARS-CoV-2. Next, we determined changes histological and cytological changes occurred in bone marrow. We detailed the progression of SARS-CoV-2 infection, and compared histology pattern and computed tomography in lung and other organs.

## 2. Methods

### 2.1. Virus

Delta (B.1.617.2) variants of SARS-CoV-2 [18] were propagated in Vero and VeroE6 cells in medium with Eagle, -MEM (Sigma) containing 10% fetal bovine serum at 37 °C. All experiments with SARS-CoV-2 were performed in enhanced biosafety level 3 (BSL3). SARS-CoV-2 from the nasal and oral mucosa was determined by means of biological titration in Vero E6 cells. Synonymous data was obtained using qRT-PCR.

### 2.2. Cells

Vero and Vero E6 cells were cultured in Eagle medium (Sigma) with 10% of inactivated fetal bovine serum and an additional 2 mM L-glutamine and 1 mM sodium pyruvate, and cells were seeded at 2 × 10^5^ cell/mL.

To investigate replication characteristics of SARS-CoV-2Delta strains, Vero cells were infected with virus at a 0.1 TCD_50_/mL. Cell lysates obtained after freezing and thawing was collected for virus titration at 1, 2, 3, 4, 5 days post infection (dpi). For the virus titration, Vero cells were seeded in 96-well plate. For virus quantification Vero and Vero E6 cells were seeded in 96 well plates and after infection were examined daily for presence of a distinguishable cytopathic effect, and the final reading was made after 9 days. The virus titer was calculated according to Reed and Muench method. Control wells containing uninfected cells. The virus concentration was expressed as TCD_50_/mL.

### 2.3. Quantitative RT-PCR

TaqMan Real-time PCR assay was performed on an Eco Illumina Real-Time PCR system device (Illumina Inc. Headquartered in San Diego, CA, USA), according to [19]. The final reaction volume consisted of 10 μL PCR reaction mix (Vazyme Ltd., Bangkok, Thailand), 1.5 μL primer/probe mix (ORF-F: 5′−CCCTGTGGGTTTTACACTTAA-3′ (2 μM final concentration); ORF-R: 5′-ACGATTGTGCATCAGCTGA-3′ (2 μM final concentration); ORF-P: 5′-FAM−CCGTCTGCGGTATGTGGAAAGGTTATGG-BHQ1−3’ (0.5 μM final concentration); N-F: 5′-GGGAGCCTTGAATACACCAAAA-3′ (2 μM final concentration); N-R: 5′-TGTAGCACGATTGCAGCATTG-3′(2 μM final concentration); N-P: 5′-HEX-AT CACATTGGCACCCGCAATCCTG-BHQ2–3’ (0.5 μM final concentration)), 5 μL template (sample, negative or positive control) was added to the final reaction volume of 20 μL.

Thermal cycling was performed by described protocol [19]. Samples were considered positive when a signal was detected at Ct < 40 for any gene.

For alignment of the cDNA plots, Cq values were rescaled after comparing with viral genome copies amounts and modified in absolute amounts along the y-axis for better visualization.

### 2.4. Nanopore Sequencing

Nanopore sequencing was performed according to “nCoV-2019 sequencing protocol v3 (LoCost) V.3” [20] based on ARTIC SARS-CoV-2 sequencing protocol with ARTIC nCoV-2019 V3 PCR panel (https://artic.network/nCoV-2019, accessed on 19 October 2022). Base calling and demultiplexing were performed using Guppy (v4.0.14). Raw FASTQ files were filtered and reads with length 400–700 b were selected using ARTIC pipeline (release 1.1.0) (Artic Network, n.d.). Downstream analyses were performed using the nanopolish workflow implemented in the ARTIC pipeline [21]. The pipeline includes an alignment to the hCoV-19/Wuhan/WIV04 reference genome with minimap2 (2.17-r941) [22] followed by variant calling and consensus-building. Positions in consensus genomes with CoVerage lower than 20 were masked with “N” bases.

Origin Virus and Virus after adaptation complete genomes were aligned using Clustal Omega (https://www.ebi.ac.uk/Tools/msa/) accessed on 1 January 2022 and similar algorithms, such as Muscle and Mafft (general purpose multiple sequence alignment programs for DNA sequences) for detection genes regions with SNPs.

### 2.5. Staining

For the analysis of cellular pathology, Vero cells were grown on glass, then infected by SARS-CoV-2, then fixed and stained with Heidenhain’s iron hematoxylin (0.5%; Sigma-Aldrich, (Merck KGaA, Darmstadt, Germany) and Mayer’s hematoxylin (0.1%; Sigma-Aldrich, Germany).

### 2.6. Experimental Infection of Syrian Hamsters

Seven to nine month old male Syrian hamsters (*n* = 36) were used in this study. Baseline body weights were measured before infection.

Under ketamine−xylazine anesthesia, 32 hamsters were inoculated with 10^6^ TCD50/mL (in 110 μL) via a combination of the intranasal (100 μL) and ocular (10 μL) routes. Four hamsters were used as control group. Body weight was monitored daily for 14 d. Blood from the gingival vein was used for daily blood smears [10]. For pathological examinations, four hamsters at 2, 5, 7, 9, 12, 16, 18, 21 days post infection were euthanized with a lethal dose of sodium thiopental, and their organs (nasal mucosa, lungs, liver, kidney, bone marrow, lymphatic nodes, spleen and blood) were collected. Syrian hamsters with SARS-CoV-2 infection were conducted in Animal Biosafety Level 3 facilities at Institute of Molecular Biology.

### 2.7. Bone Marrow Imprints and Cell Analysis

Syrian hamsters were euthanized with a lethal dose of sodium thiopental. Hamsters for bone marrow investigations were euthanized at 2, 7, 9, 18 and 21 days postinfection (dpi) in batches of four. Fresh spleen and lymph nodes (from the abdominal cavity) were cut with a sharp blade through the hilum, and tissue imprints were made by gently touching the freshly cut surface of the tissue with a clean glass microscope slide. For bone marrow, the needle was advanced in the femoral bone marrow cavity with a twisting motion and rotated to obtain a solid piece of bone marrow, after which the imprints were made. The remaining Syrian hamsters (control group) were euthanized as shown above.

For blood cell analysis, slides were fixed in pure methanol and stained via the Pappenheim method according to the manufacturer’s protocol (Sigma-Aldrich, Germany). Cells were analyzed and counted in 100 randomly selected fields (0.01 mm^2^) using a light microscope at 1250 magnification. In total, at least 3000 cells were analyzed and classified at each time point of infection.

### 2.8. Tissue Samples

Samples from lung, liver, kidney, lymph nodes and other organs in 10% buffered formalin solution (pH 7.2) for 24 h. After fixation, the samples were dehydrated through a graded series of alcohols, washed with xylol and embedded in paraffin wax by a routine technique for light microscopy. For structural analysis, wax-embedded samples were cut (Microm HM 355, 5 lm) and stained with hematoxylin and eosin according to the manufacturer’s protocol (Sigma-Aldrich, Germany). The histological examination was carried out using a light microscope as described previously [23].

### 2.9. Micro-CT Imaging

All micro-CT imaging was performed using micro-CT system Bruker SkyScan 1276 Micro-CT. Animals were scanned while breathing freely under anesthesia using standard ketamine−xylazine anesthesia. Micro CT according to [10] was used to qualitative and semiquantitative image evaluation of the lung abnormalities by using a CT severity score adapted from a human scoring system.

### 2.10. Statistical Analysis

All in vitro experiments were conducted in triplicate. The significance of virus-induced changes was evaluated by two-tailed Student’s t-test for parametric values and Mann-Whitney u-test for non-parametric values; *p* values < 0.05 were considered significant. SPSS version 17.0 software package (SPSS Inc., Chicago, IL, USA) was used for statistical analyses.

## 3. Results

### 3.1. Isolation and Adaptation of SARS-CoV-2Delta Variant

The virus was isolated from nasopharyngeal swabs of 11 patients with a confirmed diagnosis of SARS-CoV-2 (RT-PCR). First 24 h monolayer of Vero cells was used for adaptation of SARS-CoV-2 derived from a positively testing nasopharyngeal swab of patient. Virus contained nasopharyngeal swabs were added to the cells for 1 h at 37 °C, after which cells were washed twice with PBS and maintained in Eagle’s minimal essential medium (MEM; Sigma). Viral titres were determined both by biological titration and RT-PCR. Below are the data of one of the isolates isolated from patient G. Virus isolation was carried out several times, options were used both without freezing/thawing, and with freezing thawing, up to three times the procedure. After one or two blind passages in the Vero cell culture, the virus began to stably demonstrate a cytopathic effect. The most successful adaptation of the virus was observed with a single freezing/thawing of cell lysates from a blind passage. After adaptation of the virus to Vero cells, we compared the features of SARS-CoV-2 replication in Vero and Vero E6 cells. In this case we did not reveal significant differences between the basic features of viral replication (replication kinetics, infectious titer, etc.) between them. It is important to note that we obtained a similar result of viral replication when adapting SARS-CoV-2 alpha strain circulating in Armenia to Vero and Vero E6 cells (unpublished data).

### 3.2. Cytopathic Effect

**Figure 1:**
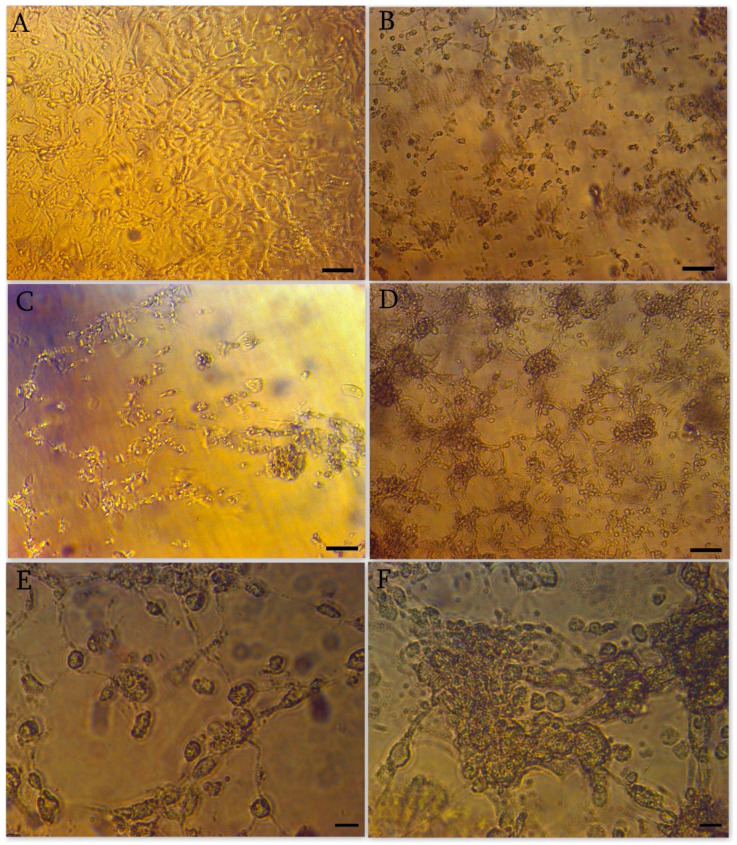
Cytopthic effect of SARS-CoV-2 on unstained Vero E6 cells. (**A**) Mock-infected control cells; scale bar 100 µm. (**B**) Cell size reduction, cell rounding 96 hpi; scale bar 100 µm (**C**) Cells deattachment, and syncytium formation; 120 hpi scale bar 100 µm. (**D**) Cells rounding 120 hpi; scale bar 10 µm. (**E**) Cells rounding, deattachment, and start of syncytium formation 120 hpi; scale bar 10 µm. (**F**) Syncytium formation, 120 hpi; scale bar 10 µm.

**Figure 2:**
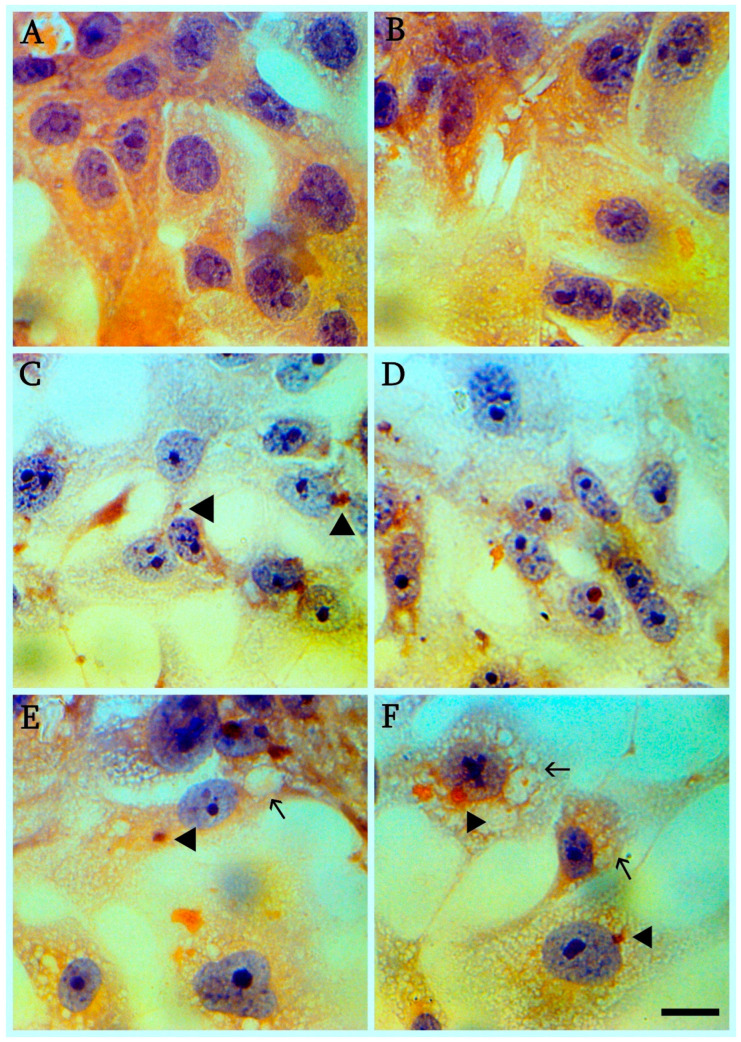
Cytopthic effect of SARS-CoV-2 on hematoxilin-eosin stained Vero E6 cells (120 hpi). (**A**) Mock-infected control cells (**B**) Mock-infected control cells (**C**) Cell size reduction, eosinophylic viral assemblies in infected cells (shown with triangle). (**D**) Cells size reduction and separation of cells from each other. (**E**) Eosinophylic viral assemblies in infected cells (shown with triangle) and vacuolization of the cytoplasm (arrowed). (**F**) Simultaneous vacuolization (arrowed) and formation of viral assemblies in same cell (shown with triangle); scale bar for (**A**–**F**) 10 µm.

The first cytopathic effect on Vero cells (Figure 1A—mock-infected control) is usually observed 48–72 h post infection (hpi). The most pronounced cytopathic effect is detected by 96–120 hpi. Typical cytopathic effects in Vero cells include cell size reduction, cell rounding, deattachment, and syncytium formation (Figure 1B–F). Hematoxylin-eosin staining reveals significant changes in virus-infected cells (Figure 2C–F) compared to control (Figure 2A,B). It is important to note that even at the late stages of viral infection in the affected cells the nucleoli are preserved. (Figure 2C,D) in infected cells is possible to detect vacuolization of the cytoplasm and viral assemblies (Figure 2E,F).

**Figure 3:**
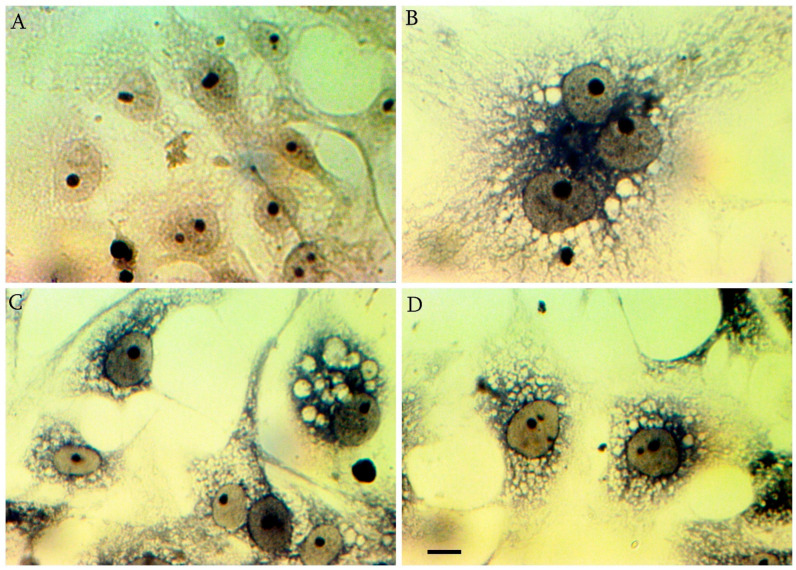
Cytopathic effect of SARS-CoV-2 on Iron hematoxylin stained Vero E6 cells (120 hpi). (**A**) Mock-infected control cells (**B**) The changes relate to the formation of synthicia (**C**) Massive vacuolization of infected cells (**D**) Vacuolization and disruption of the cell confluent (Figure 3D); scale bar for (**A**–**D**) 10 µm.

Iron hematoxylin staining reveals subtle changes in virus-infected cells compared to controls (Figure 3A). The changes relate to the formation of syncytia (Figure 3B), vacuolization (Figure 3C) and disruption of the cell confluent (Figure 3D).

### 3.3. SARS-CoV-2 Quantification In Vitro

**Figure 4:**
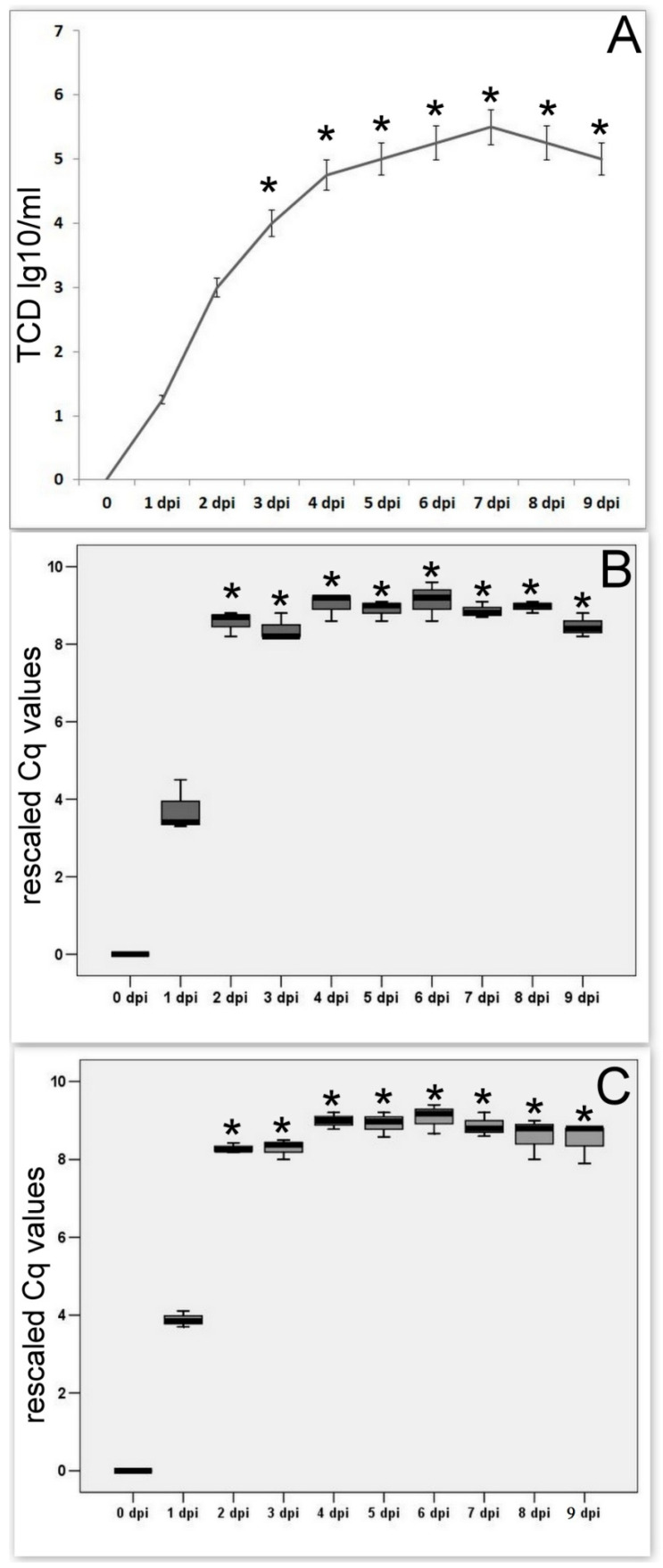
SARS-CoV-2 quantification in vitro. (**A**) Biological titration of SARS-CoV-2 on Vero E6 cells. (**B**) qRT-PCR detected levels of Nucleocapsid (N) Gene copies of SARS-CoV-2on Vero E6 cells. (**C**) qPCR levels of ORF1 copies of SARS-CoV-2 on Vero E6 cells.

Infectious titers of the virus, immediately after adaptation, starting from the 2nd passage, demonstrated stable levels, and varied within 10% Figure 4. The Figure 4A shows that SARS-CoV-2 titers (detected by biological titration) on Vero E6 cells are gradually increasing and reached a maximum by 4–7 days after infection. The number of copies of the virus genomes (Figure 4B,C) detected using qRT-PCR reaches a maximum by 48 hpi (earlier compared with biological titration) and then stably remains at maximum values (approximately 8–9 thousand copies per 20 μm).

### 3.4. Changes in the SARS-CoV-2 Delta Variant Genome at Adaptation to Replication in VERO Cell

In our study, we used viral RNA sequencing to detect changes in the frequency of transition mutations during adaptation to Vero cell culture. The obtained results show the greatest number of mutations was found in the ORF1ab, S and N genes, which products are RNA dependent RNA polymerase (RDRP), surface glycoprotein and nucleocapsid phosphoprotein (Table 1).

### 3.5. Changes in Blood Cell Populations in Syrian Golden Hamsters

**Figure 5:**
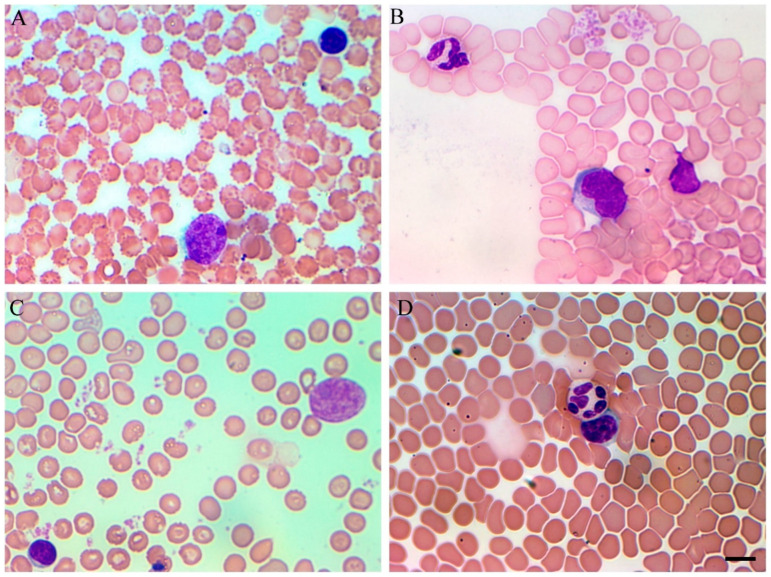
Blood smears in SARS-CoV-2 infected Syrian hamsters (Giemsa stained). (**A**) Lymphoblasts at 2nd dpi (finely expressed nucleoli inside the nucleus) (**B**) Monoblast at 1st dpi. (**C**) Myeloid cell at 3rd dpi. (**D**) Hypersegmented neutrophil at 3rd dpi. Scale bar for (**A**–**D**) 10 µm.

SARS-CoV-2 in hamsters is characterized by dynamic but not very pronounced decrease of all the nuclear blood cells. The percentage of metamyelocytes increased compared to the control values and early precursors myeloid cells appear. In the lymphoid population, there is a decrease in the number of mature cells, both absolute and relative. Simultaneously in lymphoid cell population increased number of lymphoblasts (finely expressed nucleoli inside the nucleus Figure 5A) and activated lymphocytes with changed nucleo-cytoplasmic ratio. Similar processes were observed in monocyte population when the monoblasts (Figure 5B) differentiated since 1st dpi, followed by decrease of total count of both monocytes and monoblasts. Along with SARS-CoV-2 progressing early blast cell forms developed. The myeloid population that normally presented with mature neutrophils (bands and segmented), and few metamielocytes developed increase in metamyelocytes population starting from 1st dpi (Figure 5C). This phenomenon quickly recovers and already by 5 dpi exceeds the benchmarks. A similar effect is revealed until the end of the study period at 9 dpi. Simultaneously with the increase in the percentage of the myeloid population, we revealed a significant shift to the left.

Along with early stages of cells, pathological cells (such as hypersegmented neutrophils—Figure 5D) also appear in the blood of SARS-CoV-2 infected animals (Table 2).

### 3.6. Changes in Bone Marrow Cell Populations

**Figure 6:**
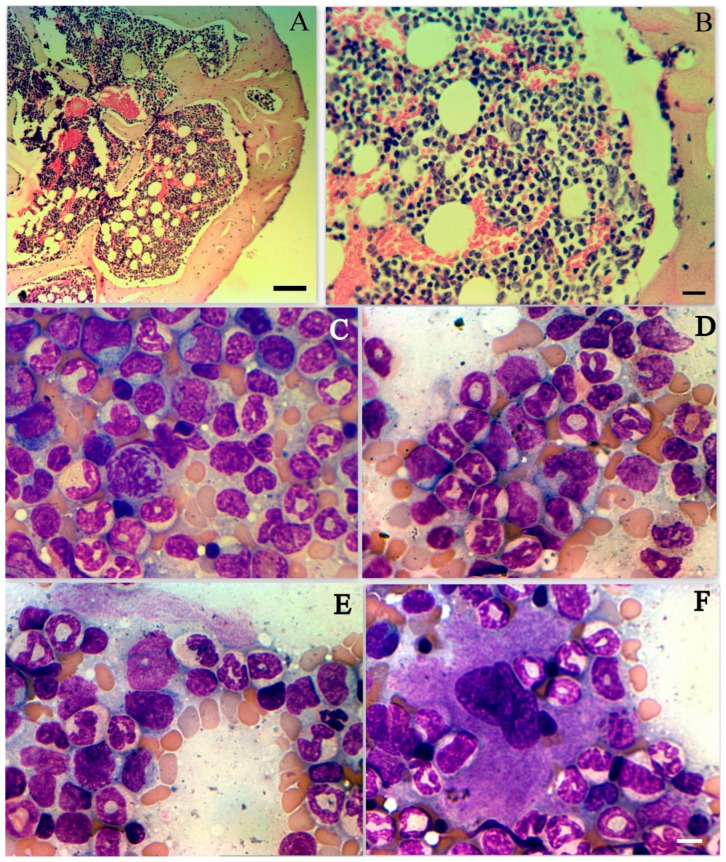
SARS-CoV-2 infection in hamster bone marrow. (**A**) Preserved structure of BM in SARS-CoV-2 infected hamsters bone marrow structure preserved (3rd dpi); HE staining; scale bar 250 µm. (**B**) Normal cellularity in BM in SARS-CoV-2 infected hamsters (3rd dpi); HE staining; scale bar 100 µm. (**C**) Decreased number of nucleated erythroid cells on 5th dpi in BM. Giemsa staining; Present mitosis; scale bar 10 µm. (**D**) Decreased number of nucleated erythroid cells on 7th dpi in BM. Giemsa staining; scale bar 10 µm. (**E**) Decreased number of nucleated erythroid cells on 8th dpi in BM. Giemsa staining; scale bar 10 µm. (**F**) Preserved megakaryocyte on 7th dpi in BM. Giemsa staining; scale bar for (**A**–**F**) 10 µm.

In SARS-CoV-2 infected hamsters bone marrow structure preserved (Figure 6A) BM sections usually were normocellular, sometimes few lowcellular (Figure 6B).

The cell population in bone marrow of uninfected animals was mainly represented by nucleated erythroid cells as well as myeloid cells (predominantly myeloid cells, mtamyelocytes and band neutrophils) constituting about 90% of the total cells (Table 3).

Upon SARS-CoV-2 infection, the number of nucleated erythroid cells significantly decreased and reached its minimum value (bout 20%) at 9 dpi (Figure 6C–F). A decrease in the population occurs due to a decrease in the content of all forms of erythroblasts. In contrast, increases in the number of lymphoid cells as well as monoblasts and monocytes. In the myeloid population, there is a decrease in early cell forms (myeloid cells and metamyelocyes) and an increase in the content of neutrophils (Table 2). It should also be noted an increase in the number of resident macrophages, megakaryocytes and an increase in the number of erythroblastic islets. We also found an increase in the proliferative activity of bone marrow cells, which manifests itself in an increase in the number of mitoses (Figure 6C). SARS-CoV-2 infection caused the formation of pathological forms, represented by atypical lymphocytes and neutrophils.

### 3.7. SARS-CoV-2 Infection in Syrian Golden Hamsters

**Figure 7:**
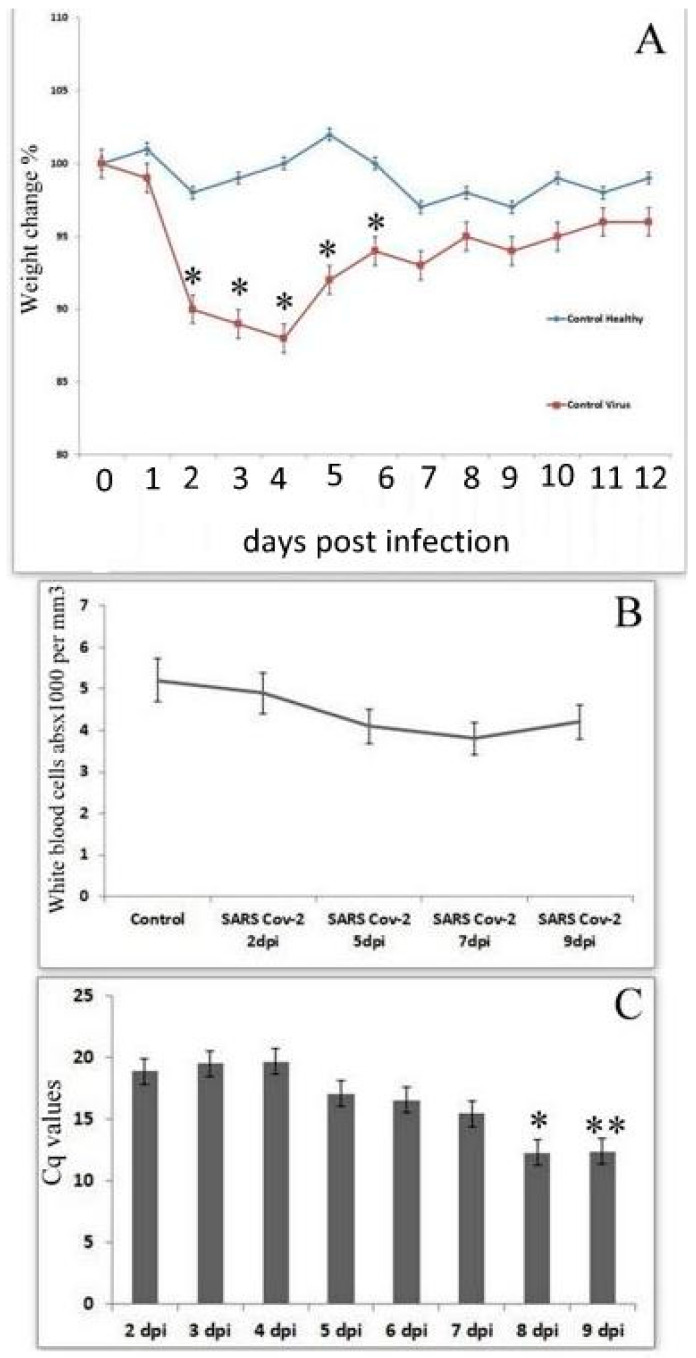
Physiological indices and virus presence in SARS-CoV-2 infected hamsters. (**A**) Dynamics of the body weight in SARS-CoV-2 infected hamsters. (**B**) Dynamic in absolute number of white blood cells in SARS-CoV-2 infected hamsters. (**C**) Viral load in oronasal swabs during SARS-CoV-2 infection in Syrian hamsters. * Significant compared with corresponded titers from groups treated with nucleotide analogues (*p* < 0.05–*p* < 0.01), ** tendency (*p* < 0.1).

Upon infection with SARS-CoV-2 (Delta variant), the first clinical symptoms were observed at 2–3 dpi, when all animals demonstrated loss of body weight and development of interstitial pneumonia. At SARS-CoV-2 infection hamsters showed only modest weight loss (7–12%) in stages 2–3 to day 7 post infection (Figure 7A). A slight rise in temperature was noted in a small number of hamsters (10–15%). Analysis of nuclear blood cells revealed a tendency towards a decrease in the total amount of WBC within 5–7 dpi (Figure 7B).

The virus was isolated in nasopharyngeal washes from all experimentally infected animals up to 7 days after infection. Then the virus disappeared in half of the studied hamsters (Figure 7C), and at later stages the replication of the virus disappear from the hamsters. Virus titers in all studied animals during the entire experiment did not demonstrate the dynamics of growth or decline.

### 3.8. Lung Computed Tomography and Gross Pathology

**Figure 8:**
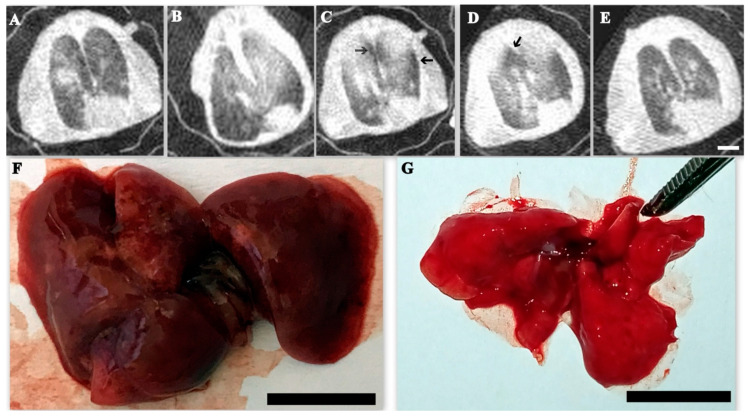
Lung computed tomography and Gross pathology in SARS-CoV-2infected hamsters. (**A**) Local ground-glass opacities with a central, peribronchial distribution in SARS-CoV-2 infected animals (3rd dpi). (**B**) Ground-glass opacities mixed with areas of focal consolidation (4th dpi). (**C**) Local emphysema formation (arrowed) at 5th dpi. (**D**) Massive emphysema formation (arrowed) at 7th dpi (**E**) Scale bar 0.5 cm for (**A**–**E**) Recovering at 12th dpi and residual, minimal lung abnormalities (**F**) Lung autopsy (SARS-CoV-2 infected hamsters on 7 dpi), the lungs are enlarged (scale bar 1 cm), flabby consistency. Many local hemorrhages. (**G**) Gross pathology study did not reveal any visible pathology in all studied hamsters since 18th dpi. (scale bar 1 cm).

First pathological patterns aroused in SARS-CoV-2 infected animals are local ground-glass opacities with a central, peribronchial distribution (Figure 8A).

Then (at 4–5 dpi) lung abnormalities have pronounced progress to more severe, peripherally distributed, rounded, ground-glass opacities mixed with areas of focal consolidation (Figure 8B). The most severe lung abnormalities occurred 5 d to 9 d post infection and developed severe pulmonary damage with high percentage of affected tissue as well as microp3ulmonary rupture and local emphysema formation (Figure 8C,D)

Improvement of CT lung pathologies starts since in some animals 7 d to 10 d postinfection with gradual decrease in ground-glass opacities. Residual, minimal lung abnormalities were present at 12 dpi (Figure 8E).

At autopsy (SARS-CoV-2 infected hamsters on 7 dpi), the lungs are enlarged, flabby consistency (Figure 8F). Gross pathology investigations during autopsy also revealed local hemorrhages were aroused starts day 3–5 dpi. By 9–12 days after infection, this lung pathology sharply decreases and by 18 days after the disease, the Gross pathology study did not reveal any visible pathology in all studied hamsters (Figure 8G).

### 3.9. Inner Organs Histopathology

**Figure 9:**
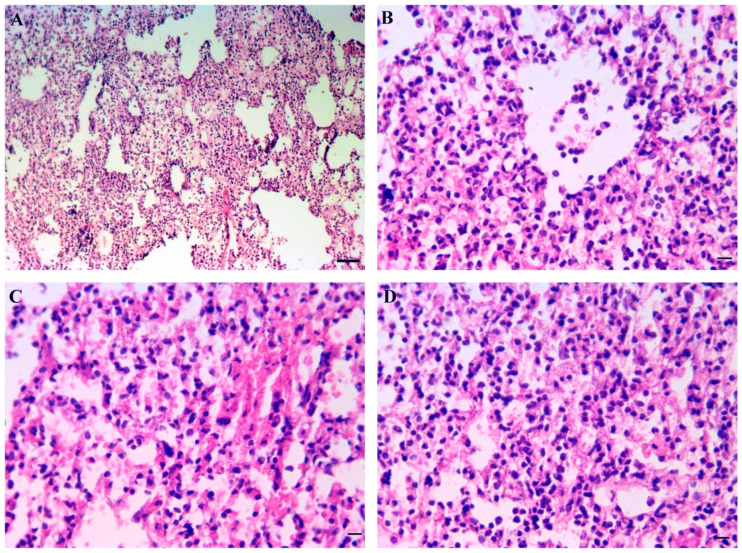
Lung pathology in hamsters after infection by SARS-CoV-2 (Delta strain). (**A**) Diffuse lymphocytic-macrophage infiltrates, acute emphysema with rupture of the interalveolar septa (5 th dpi) HE staining; scale bar 100 µm. (**B**) Diffuse lymphocytic-macrophage infiltrates, acute emphysema with rupture of the interalveolar septa (5 th dpi) HE staining; scale bar 50 µm. (**C**) Pronounced hyperemia of the vasculature of the interalveolar septa at 9th dpi HE staining; scale bar 50 µm. (**D**) Focal hemorrhages are noticeable; in the lumens of some alveoli, cellular infiltrates are found at 9th dpi HE staining; scale bar 50 µm.

Against the background of moderately pronounced edema of the interstitial tissue (Figure 9A), diffuse lymphocytic-macrophage infiltrates are found. In some alveoli, acute emphysema with rupture of the interalveolar septa is determined (Figure 9A,B). At SARS-CoV-2 infection on 9 dpi developed pronounced hyperemia of the vasculature of the interalveolar septa. Focal hemorrhages are noticeable; in the lumens of some alveoli, cellular infiltrates are found (Figure 9C,D). The peak of histological changes in the lungs of hamsters falls on the 5–9th day from the onset of the disease, and then there is a rapid spontaneous restoration of the normal picture of the lung tissue.

**Figure 10:**
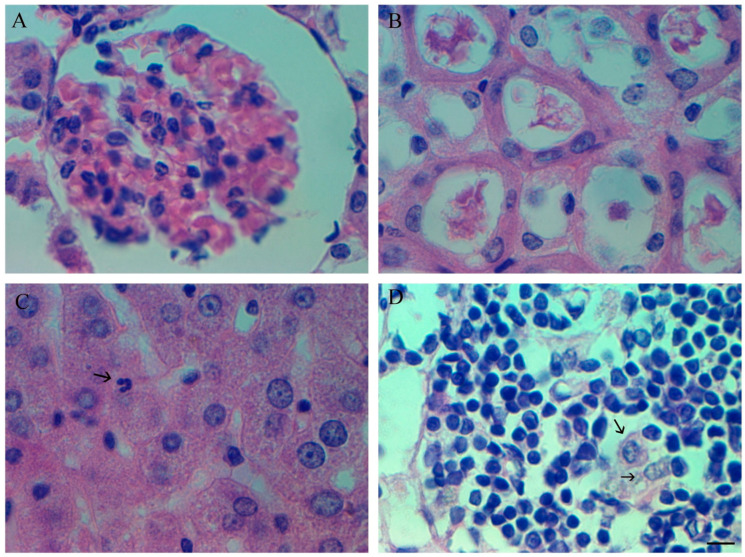
Inner organs pathology after infection by SARS-CoV-2 (Delta strain). (**A**) Hyperemia of glomeruli (5th dpi) HE staining; scale bar 10 µm. (**B**) Hyaline masses visible in the proximal canals (5th dpi) HE staining; scale bar 10 µm. (**C**) Light neutrophilic diapedesis in liver (5th dpi) HE staining; scale bar 10 µm. (**D**) Increased number of resident macrophages in lymph nodes increases (7th dpi) HE staining; scale bar 10 µm for (**A**–**D**).

In the kidneys oh infected hamsters during histological examination, there are practically no pathological a change throughout the experimental infection, only some hyperemia of glomeruli is noted (Figure 10A). The cytoangioarchitectonics of the kidneys is preserved, there is no edema and hemorrhages hyaline masses are visible in the proximal canals, however, a similar pattern was observed in healthy hamsters (Figure 10B).

The liver has no visible changes, and microscopic examination of tissues reveals only isolated cases of neutrophilic diapedesis (Figure 10C). However, such signs are possible in a healthy liver of hamsters.

Histological examination of the lymph nodes and spleen revealed a tendency towards a change in the cell population (a slight decrease in the lymphocyte content, and an increase in the myeloid population—unpublished data. At the same time, starting from 5–7 dpi up to 10–12 dpi, the content of resident macrophages in lymph nodes increases (Figure 10D).

## 4. Discussion

Adaptation of the virus to replication in vitro is not difficult and does not require the use of additional techniques [6]. Changes in the virus genome expressed by the single nucleotide polymorphism in different genes are the initial stage during the transition to replication in cell culture.

Susceptibility of Syrian hamsters to SARS-CoV-2 virus has been shown in a number of articles [24,25,26]. SARS CoV-2 induces typical severe lung abnormalities mostly occurred 5 d to 9 d post infection with high percentage of affected tissue as well as micropulmonary rupture and local emphysema formation. Unlike the delta strain, the Omicron strain does not cause serious changes in the lungs of hamsters, and the disease proceeds in a mild form within 2–4 days (unpublished data). Clinical manifestations and main symptoms of the SARS-CoV-2 infection on Syrian hamsters model during involvement in COVID-19 disease remains uncertain [27]. Some studies have shown that hamsters with COVID-19 had liver dysfunction [13,16]. However, our data, like data of [12,24,26] showed that the liver had no visible changes. Although in our experiments isolated cases of neutrophilic diapedesis were detected.

As previously documented [28], kidney damage in case of Syrian hamsters is not caused by direct viral infection or replication and is depended on age of examined hamsters. We also report that the hamster kidney does not undergo significant pathological changes during SARS-CoV-2 infection.

The changes revealed in the peripheral blood generally correspond to the pathology in the inflammatory process. The rejuvenation of the leukocyte population and its rapid recovery indicate the compensatory nature of these changes. The appearance of pathological lymphocytes is most likely not associated with the direct pathological effect of the virus, but with accelerated cell proliferation. Similar processes have been described in the bone marrow of SARS-CoV-2 infected hamsters. For the erythroid population, not only the washing out of early forms (erythroblasts) into the peripheral blood is characteristic, but also a change in the population of erythroblasts in the bone marrow. Changes in the myelogram and in the analysis of erythroblastic islets revealed an acceleration of erythropoietic processes. It should be noted that such changes occur without hemolysis, therefore, the reasons for the acceleration of erythropoiesis in the bone marrow are not entirely clear. This can be explained both by compensatory processes and by disturbances in erythropoiesis caused by a viral infection.

## 5. Conclusions

Thus, we have described the main characteristics of the pathogenesis of the SARS Cov-2 virus both in cell culture and in susceptible animals. Data on the pathogenesis caused by a virus in hamsters suggests the presence of a systemic disease affecting all internal organs. The data obtained by us allow us to determine the main criteria of pathogenesis and the dynamics of their changes, the features of virus replication under conditions of experimental infection.

## Figures and Tables

**Table 1:** The SNPs in genes obtained by sequencing the SARS-CoV-2 virus adapted on Vero cells.

Positions	Virus	Gene, Product	Sequence
809	Original virus Virus after early stage of adaptation on Vero cells	gene = “ORF1ab product = “nsp2	AGGGCATACACTC AGGGCACACACTC
14,790	Original virus Virus genome after adaptation on Vero cells	gene = “ORF1ab product = “RNA-dependent RNA polymerase	TGCTATCAGCGAT TGCTATTAGCGAT
14,925	Original virus Virus genome after adaptation on Vero cells	gene = “ORF1ab product = “RNA-dependent RNA polymerase	CATCGTTAACAAC CATCGTCAACAAC
15,952	Original virus Virus genome after adaptation on Vero cells	gene = “ORF1ab product = “RNA-dependent RNA polymerase	AGAATCATAGGGG AGAATCCTAGGGG
18,117	Original virus Virus after adaptation on Vero cells	gene = “ORF1ab” product = “3’-to-5’ exonuclease	TACACATCTCAGT TACACACCTCAGT
18,744	Original virus Virus genome after adaptation on Vero cells	gene = “ORF1ab” product = “3’-to-5’ exonuclease	TGATTATGTCTAT TGATTACGTCTAT
21,137	Original virus Virus genome after adaptation on Vero cells	gene = “ORF1ab product = “2’-O-ribose methyltransferase	AACAAAGGCTAGC AACAAAAGCTAGC
23,803	Original virus Virus after adaptation on Vero cells	Gene S spike glycoprotein product surface glycoprotein	TTCAACCGAATGC TTCAACTGAATGC
24,410	Original virus Virus after adaptation on Vero cells	Gene S spike glycoprotein product surface glycoprotein	GACCTCCTTTGTG GACCTCATTTGTG
25,117	Original virus Virus after adaptation on Vero cells	Gene S spike glycoprotein product surface glycoprotein	TGACCGTCTCAAT TGACCGCCTCAAT
25,439	Original virus Virus after adaptation on Vero cells	gene= ORF3a product ORF3a protein	CTTTGACGCAAGG CTTTGAAGCAAGG
25,913	original virus Virus after adaptation on Vero cells	gene = ORF3a product ORF3a protein	GTGATGACACAAC GTGATGGCACAAC
26,885	original virus Virus after adaptation on Vero cells	gene = M product membrane glycoprotein	TCTCAATGTGCCA TCTCAACGTGCCA
28,299	original virus Virus after adaptation on Vero cells	gene = N product = “nucleocapsid phosphoprotein	AAAATCTGCGAAA AAAATCAGCGAAA
28,697	original virus Virus after adaptation on Vero cells	gene = N product = nucleocapsid phosphoprotein	AATACATCAAAAG AATACACCAAAAG
28,748	original virus Virus after adaptation on Vero cells	gene = N product = nucleocapsid phosphoprotein	ATCGTGTTACAAC ATCGTGCTACGAC

**Table 2:** Blood cells populations in norm and under the action of SARS CoV-2.

Cells	Control	SARS-CoV-2-22 dpi	SARS-CoV-25 dpi	SARS-CoV-27 dpi	SARS-CoV-29 dpi
Erythroblast	-	0.9 ± 0.1	-	-	-
Lymphoblast	0.3 ± 0.1	3.2 ± 0.5 *	2.7 ± 0.5 *	2.0 ± 0.3	3.0 ± 0.4 *
Lymphocyte	61.8 ± 7.1	54.7 ± 6.3	44.0 ± 4.9	30.9 ± 4.2 *	46.9 ± 5.1
Limph.patol.	-	1 ± 0.1 *	1.0 ± 0.1 *	1.0 ± 0.1 *	-
Monoblast	0.4 ± 0.1	1 ± 0.1	-	1.0 ± 0.1	1.0 ± 0.1
Monocyte	3.9 ±0.4	2.8 ± 0.2	3.3 ± 0.6	4.0 ± 0.5	3.0 ± 0.2
Mieloid	-	3.0 ± 0.6 *	0.01 ± 0.01	2.0 ± 0.4 *	1.0 ± 0.3
Metamielocyte	1.4 ± 0.1	3.2 ± 0.2	0.1 ± 0.04	3.3 ± 0.5	3.0 ± 0.4
Band	17.5 ± 2.6	15.0 ± 2.7	30.3 ± 3.0	30.2 ± 2.8 *	25.7 ± 2.2
Segment	13.3 ± 2.1	10.5 ± 1.8	15.3 ± 1.3	22.5 ± 1.9 *	15.0 ± 1.5
Pathol neutrophil	-	2.0 ± 0.7 *	0.01 ± 0.01	3.0 ± 0.9 *	0.5 ± 0.7
Eosinophil	1.4 ± 0.2	1.5 ± 0.3	1.0 ± 0.1	0.1 ± 0.1	0.5 ± 0.2
Basophil	0.01 ± 0.01	0.01 ± 0.01	0.3 ± 0.1	0.01 ± 0.01	0.01 ± 0.01
Destroied	-	1.0 ± 0.05 *	2.0 ± 0.01 *	0.01 ± 0.01	-

* Significant (*p* < 0.01, - *p* < 0.05) compared to control.

**Table 3:** Bone marrow cell populations in norm and under the action of SARS-CoV-2.

Cellls	Control	SARS-CoV-2 9 dpi	SARS-CoV-2 18 dpi
Proerythroblast	2.09 ± 0.5	2.27 ± 0.7	1.85 ± 0.4
Basophil erythroblast	9.97 ± 1.1	5.02 ± 0.8 *	6.92 ± 1.0
Polichromatophil erythroblast	15.53 ± 2.4	6.01 ± 0.8 *	10.25 ± 1.3
Ortochrom erythroblast	9.11 ± 0.9	6.36 ± 1.5	7.21 ± 1.1
Lymphoblast	1.07 ± 0.2	3.98 ± 0.7 *	2.01 ± 0.5
Lymphocyte	3.31 ± 0.7	11.09 ± 1.1 *	4.97 ± 0.7
Limph.patol.	-	0.45 ± 0.06 *	0.29 ± 0.04
Monoblast	0.81 ± 0.1	3.09 ± 0.9 *	0.88 ± 0.07
Monocyte	1.17 ± 0.3	4.57 ± 1.1 *	2.36 ± 0.5
Myeloid	13.65 ± 2.9	4.18 ± 0.9 *	5.76 ± 0.9 *
Metamielocyte	25.62 ± 4.7	11.32 ± 2.5 *	20.34 ± 4.4
Band	14.13 ± 2.1	32.64 ± 6.4 *	27.55 ± 5.8
Segment	2.35 ± 0.6	4.18 ± 1.2	7.52 ± 2.0 *
Pathol neutrophil	-	0.81 ± 0.1 *	0.53 ± 0.06 *
Eosinophil	0.91 ± 0.2	1.45 ± 0.09	1.02 ± 0.2
Basophil	0.08 ± 0.01	0.09 ± 0.01	0.02 ± 0.01
Destroied	0.01 ± 0.01	1.80 ± 0.3 *	0.24 ± 0.02
Macroph. Resident	0.14 ± 0.05	0.24 ± 0.05	0.09 ± 0.01
Macroph. Island	0.02 ± 0.01	0.09 ± 0.01 *	0.07 ± 0.01
Megacarioblast	0.01 ± 0.01	0.06 ± 0.01	0.07 ± 0.01 *
Megakaryocyte basophil	0.01 ± 0.01	0.05 ± 0.01	0.02 ± 0.01
Megakaryocyte azurophil	0.01 ± 0.01	0.04 ± 0.01	0.02 ± 0.01
Mitos	0.01 ± 0.01	0.18 ± 0.01 *	0.01 ± 0.01

* Significant (*p* < 0.01, - *p* < 0.05) compared to control.

## Data Availability

For Nanopore sequencing analysis were used (https://artic.network/nCoV-2019) (https://www.ebi.ac.uk/Tools/msa/), accessed on 19 October 2022.

## References

[1] Hu B., Guo H., Zhou P., Shi Z.L. (2021). Characteristics of SARS-CoV-2 and COVID-19. Nat. Rev. Microbiol..

[2] Wu J.T., Leung K., Leung G.M. (2020). Nowcasting and forecasting the potential domestic and international spread of the 2019-nCoV outbreak originating in Wuhan, China: A modelling study. Lancet.

[3] Lai C.C., Shih T.P., Ko W.C., Tang H.J., Hsueh P.R. (2020). Severe acute respiratory syndrome coronavirus 2 (SARS-CoV-2) and coronavirus disease-2019 (COVID-19): The epidemic and the challenges. Int. J. Antimicrob. Agents.

[4] Chen Y., Liu Q., Guo D. (2020). Emerging coronaviruses: Genome structure, replication, and pathogenesis. J. Med. Virol..

[5] Caruso A., Caccuri F., Bugatti A., Zani A., Vanoni M., Bonfanti P., Cazzaniga M.E., Perno C.F., Messa C., Alberghina L. (2021). Methotrexate inhibits SARS-CoV-2 virus replication “in vitro”. J. Med. Virol..

[6] Dalebout T.J., Zevenhoven-Dobbe J.C., Limpens R.W.A.L., van der Meer Y., Caly L., Druce J., de Vries J.J.C., Kikkert M., Bárcena M., Sidorov I. (2020). SARS-coronavirus-2 replication in Vero E6 cells: Replication kinetics, rapid adaptation and cytopathology. J. Gen. Virol..

[7] Kumar S., Thambiraja T.S., Karuppanan K., Subramaniam G. (2022). Omicron and Delta variant of SARS-CoV-2: A comparative computational study of spike protein. J. Med. Virol..

[8] Chan J.F., Zhang A.J., Yuan S., Poon V.K., Chan C.C., Lee A.C., Chan W.M., Fan Z., Tsoi H.W., Wen L. (2020). Simulation of the Clinical and Pathological Manifestations of Coronavirus Disease 2019 (COVID-19) in a Golden Syrian Hamster Model: Implications for Disease Pathogenesis and Transmissibility. Clin. Infect. Dis. Off. Publ. Infect. Dis. Soc. Am..

[9] Lee A.C., Zhang A.J., Chan J.F., Li C., Fan Z., Liu F., Chen Y., Liang R., Sridhar S., Cai J.P. (2020). Oral SARS-CoV-2 Inoculation Establishes Subclinical Respiratory Infection with Virus Shedding in Golden Syrian Hamsters. Cell Rep. Med..

[10] Imai M., Iwatsuki-Horimoto K., Hatta M., Loeber S., Halfmann P.J., Nakajima N., Watanabe T., Ujie M., Takahashi K., Ito M. (2020). Syrian hamsters as a small animal model for SARS-CoV-2 infection and countermeasure development. Proc. Natl. Acad. Sci. USA.

[11] Muñoz-Fontela C., Dowling W.E., Funnell S., Gsell P.S., Riveros-Balta A.X., Albrecht R.A., Andersen H., Baric R.S., Carroll M.W., Cavaleri M. (2020). Animal models for COVID-19. Nature.

[12] Sia S.F., Yan L.M., Chin A., Fung K., Choy K.T., Wong A., Kaewpreedee P., Perera R., Poon L., Nicholls J.M. (2020). Pathogenesis and transmission of SARS-CoV-2 in golden hamsters. Nature.

[13] Song Z., Bao L., Yu P., Qi F., Gong S., Wang J., Zhao B., Liu M., Han Y., Deng W. (2021). SARS-CoV-2 Causes a Systemically Multiple Organs Damages and Dissemination in Hamsters. Front. Microbiol..

[14] Iwasaki M., Saito J., Zhao H., Sakamoto A., Hirota K., Ma D. (2021). Inflammation Triggered by SARS-CoV-2 and ACE2 Augment Drives Multiple Organ Failure of Severe COVID-19: Molecular Mechanisms and Implications. Inflammation.

[15] Li Q., Wu J., Nie J., Zhang L., Hao H., Liu S., Zhao C., Zhang Q., Liu H., Nie L. (2020). The Impact of Mutations in SARS-CoV-2 Spike on Viral Infectivity and Antigenicity. Cell.

[16] Francis M.E., Goncin U., Kroeker A., Swan C., Ralph R., Lu Y., Etzioni A.L., Falzarano D., Gerdts V., Machtaler S. (2021). SARS-CoV-2 infection in the Syrian hamster model causes inflammation as well as type I interferon dysregulation in both respiratory and non-respiratory tissues including the heart and kidney. PLoS Pathog..

[17] Arakelyan A., Avetyan D., Karalyan Z., Akopyan S., Nikogosyan M., Khachatryan G., Sirunyan T., Gukasyan L., Zakharyan R., Muradyan N. Molecular Genetics and Pathology of COVID-19. Proceedings of the Compedium of the II International Scientific and Practical Conference on Counteraction of the Coronavirus Infection and Other Infectious Diseases.

[18] Avetyan D., Chavushyan A., Ghazaryan H., Melkonyan A., Stepanyan A., Zakharyan R., Hayrapetyan V., Atshemyan S., Khachatryan G., Sirunyan T. (2021). SARS-CoV-2 detection by extraction-free qRT-PCR for massive and rapid COVID-19 diagnosis during a pandemic in Armenia. J. Virol. Methods.

[19] Li X., Liu J., Liu Q., Yu L., Wu S., Yin X. (2020). Sheng Wu Gong Cheng Xue Bao. Chin. J. Biotechnol..

[20] Tyson J.R., James P., Stoddart D., Sparks N., Wickenhagen A., Hall G., Choi J.H., Lapointe H., Kamelian K., Smith A.D. (2020). Improvements to the ARTIC multiplex PCR method for SARS-CoV-2 genome sequencing using nanopore. bioRxiv.

[21] Loman N.J., Quick J., Simpson J.T. (2015). A complete bacterial genome assembled de novo using only nanopore sequencing data. Nat. Methods.

[22] Li H. (2018). Minimap2: Pairwise alignment for nucleotide sequences. Bioinformatics.

[23] Zakaryan H., Cholakyans V., Simonyan L., Misakyan A., Karalova E., Chavushyan A., Karalyan Z. (2015). A study of lymphoid organs and serum proinflammatory cytokines in pigs infected with African swine fever virus genotype II. Arch. Virol..

[24] Wang Z., Munster V., Jarvis M.A., Feldmann H. (2020). Defining the Syrian hamster as a highly susceptible preclinical model for SARS-CoV-2 infection. bioRxiv.

[25] Braxton A.M., Creisher P.S., Ruiz-Bedoya C.A., Mulka K.R., Dhakal S., Ordonez A.A., Beck S.E., Jain S.K., Villano J.S. (2021). Hamsters as a Model of Severe Acute Respiratory Syndrome Coronavirus-2. Comp. Med..

[26] Rosenke K., Meade-White K., Letko M., Clancy C., Hansen F., Liu Y., Okumura A., Tang-Huau T.L., Li R., Saturday G. (2020). Defining the Syrian hamster as a highly susceptible preclinical model for SARS-CoV-2 infection. Emerg. Microbes Infect..

[27] Hsu C.J., Lin W.C., Chou Y.C., Yang C.M., Wu H.L., Cheng Y.H., Liu P.C., Chang J.Y., Chen H.Y., Sun J.R. (2022). Dynamic Changes of the Blood Chemistry in Syrian Hamsters Post-Acute COVID-19. Microbiol. Spectr..

[28] Ohno M., Sasaki M., Orba Y., Sekiya T., Masum M.A., Ichii O., Sawamura T., Kakino A., Suzuki Y., Kida H. (2021). Abnormal Blood Coagulation and Kidney Damage in Aged Hamsters Infected with Severe Acute Respiratory Syndrome Coronavirus 2. Viruses.

